# Avian Influenza Virus A (H5N1), Detected through Routine Surveillance, in Child, Bangladesh

**DOI:** 10.3201/eid1508.090283

**Published:** 2009-08

**Authors:** W. Abdullah Brooks, A.S.M. Alamgir, Rebecca Sultana, M. Saiful Islam, Mustafizur Rahman, Alicia M. Fry, Bo Shu, Stephen Lindstrom, Kamrun Nahar, Doli Goswami, M. Sabbir Haider, Sharifun Nahar, Ebonee Butler, Kathy Hancock, Ruben O. Donis, Charles T. Davis, Rashid Uz Zaman, Stephen P. Luby, Timothy M. Uyeki, Mahmudur Rahman

**Affiliations:** International Centre for Diarrhoeal Disease Research, Bangladesh, Dhaka, Bangladesh (W.A. Brooks, R. Sultana, M.S. Islan, Mustafizur Rahman, K. Nahar, D. Goswami, S. Nahar, R.U. Zaman, S.P. Luby); Johns Hopkins Bloomberg School of Public Health, Baltimore, Maryland, USA (W.A. Brooks); Institute of Epidemiology, Disease Control and Research, Dhaka (A.S.M. Alamgir, S. Haider, Mahmudur Rahman); Centers for Disease Control and Prevention, Atlanta, Georgia, USA (A.M. Fry, B. Shu, S. Lindstrom, E. Butler, K. Hancock, R.O. Donis, C.T. Davis, S.P. Luby, T.M. Uyeki)

**Keywords:** H5N1, influenza, avian influenza, human, surveillance, Bangladesh, viruses, dispatch

## Abstract

We identified avian influenza virus A (H5N1) infection in a child in Bangladesh in 2008 by routine influenza surveillance. The virus was of the same clade and phylogenetic subgroup as that circulating among poultry during the period. This case illustrates the value of routine surveillance for detection of novel influenza virus.

Human infection with highly pathogenic avian influenza (HPAI) virus A (H5N1) has been associated with severe disease (pneumonia, multiorgan failure) and often with death (*1*). However, a wider spectrum of subtype H5N1 illness has been reported (*2*). The panzootic subtype H5N1 virus strains circulating among poultry and wild birds are derived from the Asian influenza (H5N1) lineage first identified in the People’s Republic of China in 1996 (*3*). HPAI (H5N1) virus was first documented in poultry in Bangladesh during March 2007; by April 2008, the virus had spread to 47 of 64 districts in Bangladesh (*4*,*5*). We report detection of a case of subtype H5N1 infection in a human in Bangladesh, which was discovered through routine outpatient influenza surveillance.

## The Study

In partnership with the government of Bangladesh, the US Centers for Disease Control and Prevention (CDC) and the International Centre for Diarrhoeal Disease Research, Bangladesh (ICDDR,B) established nationwide hospital-based surveillance to identify clusters of persons with life-threatening influenza virus infections (*6*). Additionally, ICDDR,B has had an urban field site in Dhaka (Kamalapur), where population-based respiratory disease surveillance was begun in 1998. In April 2004, influenza surveillance was initiated there in collaboration with CDC (*7*). The site and its surveillance system have been described (*8*). As part of this surveillance, a blood culture for bacterial pathogens was collected from every child with acute respiratory or febrile illness, and from 1 in 5 (20% sampling frame) a nasopharyngeal wash specimen was collected for influenza virus culture in MDCK cells, and acute-phase and convalescent-phase serum samples were collected for detecting other respiratory viruses.

On January 29, 2008, a 16-month-old boy was brought to the Kamalapur clinic with a history of 7 days of fever, 5 days of cough and rhinorrhea, and 3 days of difficult/fast breathing and loss of appetite (Table). The child’s mother described his activity and alertness status as normal. She said he had not exhibited diarrhea, convulsions, or other signs of illness. The child had been hospitalized 1 year earlier for acute watery diarrhea that resolved uneventfully. No underlying illnesses had been diagnosed, and he had not received any medications before this clinic visit. His routine vaccinations were up to date.

**Table:** History of illness in 16-month-old boy with influenza virus A (H5N1) infection, Bangladesh, 2008

Manifestation	Finding/duration, d
Initial examination	
Fever*	Yes, 7
Rhinorrhea	Yes, 5
Cough	Yes, 5
Difficult/fast breathing	Yes, 3
Nausea/vomiting	No
Anorexia	Yes, 4
Loose/watery/mucoid/bloody stool	No
Mental status/activity changes	No
Convulsions	No
Antimicrobial drugs or other medications prior to clinic visit	No
Clinical findings (day 7)	
Temperature,°C	38.1
Pulse, beats/min	124
Blood pressure, systolic/diastolic	90/50
Respiratory rate, breaths/min	40
Head, eyes, ears, nose, throat	Rhinorrhea (clear)
Chest auscultation	Clear bilaterally
Neurologic	Alert, no distress, nonfocal
Weight, kg	8.7
Weight-for-age, %†	78.3
Chest radiograph	Bilateral perihilar alveolar infiltrates (air bronchograms)

*Chief complaint and first symptom. †Based on National Center for Health Statistics reference data.

Examination showed that the child had a fever, a mildly elevated respiratory rate, and a clear nasal discharge (Table). He showed no evidence of respiratory distress, and the lungs sounded clear. Oral thrush was noted. The child weighed 8.7 kg (78th percentile for age). Blood for bacterial culture and viral serologic testing and a nasopharyngeal wash sample were collected according to accepted routine. A chest radiograph showed scattered bilateral alveolar infiltrates. Because of the duration of his fever, he received a diagnosis of suspected typhoid fever, which is endemic to this community (*9*), and was given oral amoxicillin and nystatin for oral thrush.

At a follow-up visit to the clinic on January 31, the patient was afebrile; he was in no distress and had a lower respiratory rate (36 breaths/min). His lungs sounded clear. The patient again visited the clinic on February 5 and February 10 and was afebrile on both occasions with a mildly elevated respiratory rate (38 breaths/min) and clear lungs. The mother reported that the child had had loose stools (possibly associated with amoxicillin) on February 10, but she denied that he had diarrhea. The child was never hospitalized; he completed a 14-day course of amoxicillin for suspected enteric fever and recovered fully. His blood culture was negative for any organism, and his final diagnosis was upper respiratory tract infection. On February 13, a convalescent-phase serum sample was collected as part of routine surveillance.

Culture of the child’s nasopharyngeal wash sample showed cytopathic effects consistent with influenza virus infection; an aliquot reacted to influenza virus A antiserum but not to antiserum to subtypes H1 or H3. An aliquot of the viral culture material was shipped frozen on April 22, 2008, to CDC, where the isolate was identified as HPAI (H5N1) virus by real-time reverse transcription–PCR. The clinical specimen was recultured on embryonated chicken eggs. The complete genome was sequenced (GenBank accession nos. FJ573465–FJ573472). The virus was identified as an H5N1 clade 2.2 strain on the basis of the hemagglutinin sequence (Figure). After identification of the influenza virus isolate as HPAI (H5N1) in May 2008, an epidemiologic investigation was initiated, and serum samples were collected from the child and 3 family members. The child lived with both parents and a sister in a 1-room residence. In late January 2008, the father had brought home a well-appearing live chicken from a local market located 50 m from the house. The chicken was kept on a veranda outside the child’s room. The mother slaughtered the chicken inside the bathroom while the child slept; she did not report having washed her hands before she handled the child. The waste materials from the slaughter were then stored in a tied polyethylene bag near the house entrance for 2 hours before disposal. Microneutralization assay, using the child’s subtype H5N1 isolate at CDC, demonstrated a 4-fold rise in subtype H5N1 neutralizing antibodies between the child’s January 2008 serum specimens (titer <20/20) and May 2008 (titer 160/80) serum specimens. Serum specimens from the child’s family members tested seronegative for subtype H5N1 neutralizing antibodies (the sister was away from the household in January).

**Figure:**
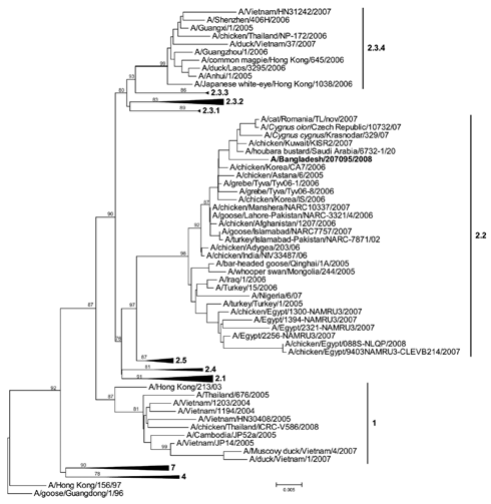
Phylogenetic tree of virus hemagglutinin sequences generated by neighbor-joining analysis. Bootstrap values at each node represent 1,000 replicates. Scale bar represents 0.005 nt substitutions. The virus found in the child, A/Bangladesh/207095/2008, is indicated in **boldface**.

## Conclusions

We report HPAI (H5N1) in a child in Bangladesh; the infection was confirmed by virus isolation from an upper respiratory specimen and by serologic testing. The source of the child’s subtype H5N1 infection is uncertain. One potential exposure was the healthy-appearing chicken that was brought inside the home. The child did not have direct contact with the chicken, although indirect contact was suggested because his mother handled him after slaughtering the chicken. The owner of the poultry shop where the chicken was purchased reported that 5%–10% of chickens had died each day during January 2008 (*10*). Because the shop was located 50 m from the home, environmental subtype H5N1 exposures cannot be ruled out. This virus was of the same clade and phylogenetic subgroup reported in poultry in Bangladesh during this period (*5*). No other household members or neighbors reported illness, and no other family members had serologic evidence of subtype H5N1 infection. In nearly 25% of reported subtype H5N1 cases worldwide, the exposure source is unclear (*2*).

In Bangladesh and other countries with influenza (H5N1) outbreaks among poultry, surveillance for human subtype H5N1 cases is focused on hospital-based case finding for febrile patients with severe acute respiratory illness. This child was not suspected of having subtype H5N1 infection and had had no known poultry contact; his illness would not have met standard criteria for subtype H5N1 testing (*11*). Instead, an upper respiratory tract specimen was collected from the child as part of routine influenza surveillance among pediatric outpatients. Similar clinically mild cases of subtype H5N1 infection in children have been identified in Turkey (*12*), Indonesia (*13*), and Egypt (*2*).

The 1 in 5 sampling frame, a major limitation of this study, raises the possibility that undetected mild cases of subtype H5N1 infection have occurred in children in this population. Other limitations include the elapsed time between illness onset and investigation and the identification of only 1 case.

The public health value of identifying the cause of severe acute respiratory illness clusters with pandemic potential is clear. This case highlights the value of routine outpatient surveillance for detecting both seasonal and novel influenza A viruses, particularly in settings in which subtype H5N1 strains circulate among poultry. Because exposure of subtype H5N1 to humans increases its opportunities for genetic mutation or reassortment, or both, with human influenza A viruses (*3*), other surveillance strategies, including cross-sectional and longitudinal serosurveys among potentially exposed persons, can help inform the extent of, and risk for, asymptomatic and clinically mild subtype H5N1 infection.
